# Nucleotide variants in hepatitis B virus preS region predict the recurrence of hepatocellular carcinoma

**DOI:** 10.18632/aging.203531

**Published:** 2021-09-17

**Authors:** Xi Chen, Minfeng Zhang, Nan Li, Rui Pu, Ting Wu, Yibo Ding, Peng Cai, Hongwei Zhang, Jun Zhao, Jianhua Yin, Guangwen Cao

**Affiliations:** 1 Department of Epidemiology, Faculty of Navy Medicine, Second Military Medical University, Shanghai, China; 2 Department of Surgery, Eastern Hepatobiliary Surgery Hospital, Second Military Medical University, Shanghai, China

**Keywords:** hepatitis B virus, viral variant, prediction model, hepatocellular carcinoma, prognosis

## Abstract

Background: Hepatitis B virus (HBV) variants in the preS region have been associated with hepatocellular carcinoma (HCC). However, the effect of the preS variants on HCC prognosis remains largely unknown. We aimed to identify the preS variants that reliably predict postoperative prognosis in HCC.

Methods: RNA-seq data of 203 HCC patients retrieved from public database were screened for the preS variants related to HCC prognosis. The variants in the sera and tumors were then validated in our prospective cohort enrolling 103 HBV-associated HCC patients.

Results: By analyzing prognosis-related gene sets in the RNA-seq data, 12 HBV preS variants were associated with HCC recurrence. Of those, G40C and C147T in the sera predicted an unfavorable recurrence-free survival in our cohort (hazard ratio [HR]=2.18, 95% confidence interval [CI]=1.37-3.47, *p*=0.001 for G40C; HR=1.84, 95% CI=1.15-2.92, *p*=0.012 for C147T). G40C and C147T were significantly associated with microscopic vascular invasion, larger tumor size, and abnormal liver function. Multivariate Cox regression analysis showed that G40C significantly increased the risk of HCC recurrence in patients with postoperative antiviral treatment. The HCC prognosis-prediction model consisting of α-fetoprotein and G40C in the sera achieved the best performance (sensitivity=0.80, specificity=0.70, and area under the curve=0.79). Functional analysis indicated that these two variants were associated with cell proliferation, chromosome instability, carcinogenesis, metastasis, and anticancer drug resistance.

Conclusions: G40C and C147T are serological biomarkers for HCC prognosis and the prognostic model consisting of serological α-fetoprotein and G40C achieved the best performance in predicting postoperative prognosis.

## INTRODUCTION

Hepatocellular carcinoma (HCC) is one of the deadliest malignant diseases. Chronic hepatitis B virus (HBV) infection is the most common cause of HCC worldwide [1]. During HBV-induced carcinogenesis, HBV keeps evolving. Some HBV mutants can promote the development of HCC [2, 3]. Due to the absence of a proofreading function for HBV polymerase, HBV has a relatively higher mutation rate during virus replication [4]. Mutations, especially mutations in the preS region of the HBV genome, are associated with advanced liver diseases, including HCC [5]. Both preS1 and preS2 deletions can cause unbalanced production of HBV envelope proteins, with consequent accumulation of the mutated large HBV surface antigen (LHBS) in the endoplasmic reticulum (ER) of hepatocytes, causing ER stress and, ultimately, HCC development [6–8]. A meta-analysis including 5563 HBV-infected patients has demonstrated that HBV preS deletion is significantly associated with an increased risk of HCC, with a summary odds ratio of 3.0 [9]. Importantly, HBV preS mutations especially deletions and some HCC-associated preS point mutations are present at least 10 years before the development of HCC [10]. These studies indicate that HBV preS mutations can predict the development of HCC in HBV-infected subjects.

The recurrence rate of HCC is high after curative resection. It is important to predict the prognosis of HCC before surgical treatment. However, reliable biomarkers for predicting HCC prognosis are lacking. Previous studies have demonstrated that higher viral load, preS deletion mutations, and higher expression of LHBS with partial pre-S2 deletion in the tumors may significantly predict the postoperative prognosis of HBV-caused HCC (HBV-HCC) cases [11–14]. However, there is no study reporting whether nucleotide variants in the preS region of HBV genome are prognostic for HCC. In this study, HBV variants in the preS region associated with postoperative prognosis of HBV-HCC were first examined by analyzing RNA-seq datasets of HCC tissues. The variants were independently validated in the sera and paired tumor tissues of HBV-HCC patients who received radical hepatectomy in a prospective cohort.

## RESULTS

### Screening of prognostic HBV preS variants in RNA-seq data

We combined the RNA-seq datasets of tumor samples of 203 HBV-HCC patients from a total of twelve studies. HBV reads could not be detected in 37 of them, while the median depth of HBV was 477.81 (IQR, 71.05–1639.56) in the remaining 166 samples. The variants at the preS region (nt. 2848 to nt. 154) covered by more than 100 reads were extracted by a procedure taking sequencing error into account. The frequencies of those variants without sufficient coverage were considered not available. To estimate the associations between HBV variants and postoperative prognosis of HCC patients, HCC-specific prognosis-related gene sets were retrieved from MsigDB and compiled into an in-house database, which contained six overall survival (OS)-related and seven recurrence-free survival (RFS)-related gene sets (Supplementary Table 1). After adjusting for any potential batch effect, sample-level enrichment scores were calculated for every gene set and correlation tests were performed. It was found that 12 HBV variants were significantly associated with RFS of HCC patients, while none was found to be associated with OS (Supplementary Table 2).

### Validation of prognostic HBV preS variants in a prospective cohort

To validate these variant-prognosis associations predicted by RNA-seq data, 103 HBV-HCC patients were enrolled in our prospective cohort (Table 1). Their sera and tumor tissues were collected and subjected to clone-based Sanger sequencing for HBV preS region. Multiple clones were sequenced for each sample, with a median clone number in the serum sample of 9 (IQR, 7–10) and in the tumor sample of 7 (IQR, 6–9). To inspect the inter-subject contamination, a heat map was plotted and indicated that there was no between-subject contamination (Supplementary Figure 1). The presence/absence of the HBV variants was summarized for each sample. Our survival analysis indicated that G40C and C147T in the tumors were significantly associated with RFS (Supplementary Table 3).

**Table 1:** Baseline characteristics of patients enrolled in our cohorts^†^.

**Variable**	**Level**	**Cohort in the study (n = 103)**
Age - yr		50.09±8.71
Gender	Male	93 (90.3)
	Female	10 (9.7)
BMI -kg/m^2^		23.50±3.75
HBV genotype (sera)	B	9 (8.7)
	C	67 (65.1)
	Mixture	27 (26.2)
Ascites	No	89 (86.4)
	Yes	14 (13.6)
Tumor rupture	No	100 (97.1)
	Yes	3 (2.9)
Portal vein tumor thrombi	No	84 (81.6)
	Yes	19 (18.4)
Tumor number	Single	85 (82.5)
	Multiple	18 (17.5)
Tumor size	<3cm	16 (15.5)
	≥3cm	87 (84.5)
Cirrhosis	No	7 (6.8)
	Mild Cirrhosis	71 (68.9)
	Cirrhosis	25 (24.3)
Tumor capsule	Complete	16 (15.5)
	Incomplete	73 (70.9)
	Absence	14 (13.6)
Microsatellite	No	74 (71.8)
	Yes	29 (28.2)
Microscopic vascular invasion	No	65 (63.1)
	Yes	38 (36.9)
Tumor differentiation	I	14 (13.6)
	II	7 (6.8)
	III	82 (79.6)
BCLC staging	0	0 (0.0)
	A	36 (35.0)
	B	48 (46.6)
	C	19 (18.4)
Postoperative antiviral treatment	No	58 (56.3)
	Yes	45 (43.7)
HBeAg	Negative	83 (70.6)
	Positive	20 (19.4)
HBV DNA - log_10_ copies/mL Total bilirubin (umol/L)		3.94±1.36
	≤20	82 (79.6)
	>20	21 (20.4)
Direct bilirubin (umol/L)	≤7	75 (72.8)
	>7	28 (27.2)
Albumin (g/L)	35-55	93 (90.3)
	<35 OR >55	10 (9.7)
AFP (ng/mL)	≤20	42 (40.8)
	>20	61 (59.2)
ALT (U/L)	≤42	55 (53.4)
	>42	48 (46.6)
AST (U/L)	≤37	48 (46.6)
	>37	55 (53.4)
GGT (U/L)	≤61	50 (48.5)
	>61	53 (51.5)
ALP (U/L)	≤129	84 (81.6)
	>129	19 (18.4)
Follow-up time (month)	Median	30.97
	IQR	11.67–59.66
HCC-related death	No	41 (39.8)
	Yes	62 (60.2)
Recurrence	No	30 (29.1)
	Yes	73 (70.9)

†Plus/minus values are means ± SD; Data are number (%), unless otherwise indicated.

ALP, alkaline phosphatase; ALT, alanine aminotransferase; AST, aspartate aminotransferase; BMI, body mass index; GGT, γ –glutamyltranspeptidase; HBV, hepatitis B virus; HCC, hepatocellular carcinoma.

**Figure 1:**
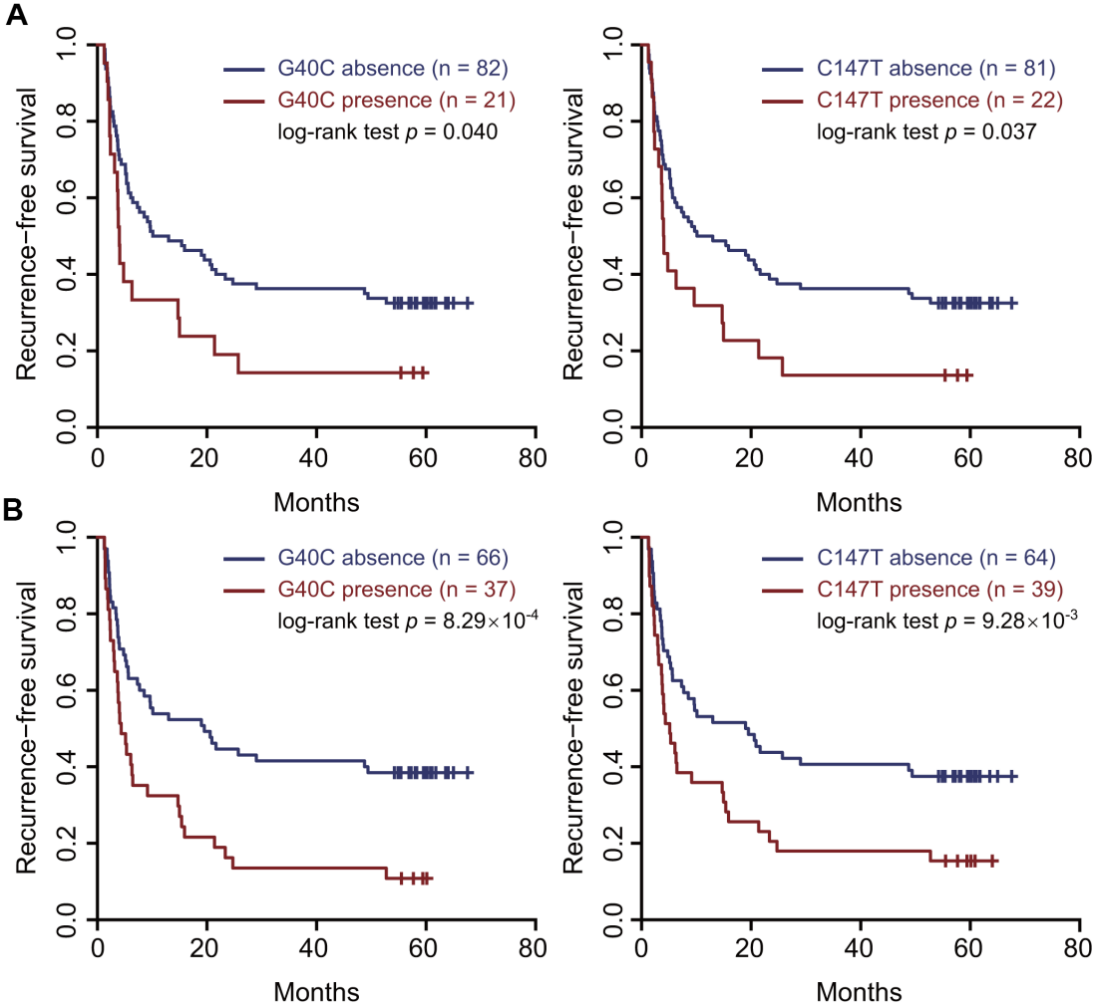
**Two HBV preS variants in the tumor tissues and sera predicted an unfavorable recurrence-free survival.** (**A**) the tumor tissues. (**B**) the sera. Patients were split into two groups according to the presence (or absence) of the variant. Kaplan–Meier curves were plotted to visualize the difference.

In the correlation test of RNA-seq, the frequencies of G40C and C147T were all associated with a gene set named “KUROKAWA_LIVER_CANCER_EARLY_RECURRENCE_UP” consisting of genes upregulated in HCC with early recurrence (r = 0.29, false discovery rate (FDR) = 0.025 for G40C; r = 0.24, FDR = 0.082 for C147T) (Supplementary Table 2). In the survival analysis of our 103 patients, the presence of these two variants in the tumors also predicted unfavorable RFS (HR = 1.78, 95% CI = 1.04–3.05, *p* = 0.045 for G40C; HR = 1.74, 95% CI = 1.03–2.95, *p* = 0.039 for C147T) (Figure 1A and Supplementary Table 3).

### Evaluation of the HBV variants as serological biomarkers for HCC prognosis

To evaluate if these HBV variants serve as serological biomarkers predicting prognosis of HCC patients, we performed survival analyses using these variants in the sera. The presence of G40C and C147T in the sera both predicted unfavorable RFS (HR=2.18, 95% CI=1.37–3.47, *p*=0.001 for G40C; HR=1.84, 95% CI=1.15–2.92, *p*=0.012 for C147T). Kaplan-Meier curves and log-rank tests also confirmed these results (Figure 1B). These two variants are polymorphic sites between the HBV genomes of HBV genotypes B2 and C2 compared to the HBV reference sequences and were highly linked. The wild types at nt. 40 and nt. 147 are G and C in genotype C2, and C and T in genotype B2, respectively. Besides, G40C does not alter amino acid, while C147T results in amino acid change from alanine to valine. By correlation tests, we found that the two variants tended to occur together, and their correlation coefficient was 0.96 in the sera. If the patients with these two variants in the sera were treated as a group, survival analysis and Kaplan-Meier curve could confirm the prediction power of two individual variants (HR=2.18, 95% CI=1.37– 3.47, *p*=0.001) (Supplementary Figure 2). Next, we investigated the associations of the combined HBV variant with clinical variables. It was found that the combined variant in the sera was significantly associated with microscopic vascular invasion (*p*<0.001), larger tumor size (*p*=0.019), and higher levels of *γ*-glutamyltranspeptidase (GGT) and alkaline phosphatase (ALP) (*p*=1.15×10^-3^ for GGT and *p*<0.001 for ALP) (Table 2). We failed to perform stratified analysis for these two variants in the sera of HBV genotype C, as these variants were barely present in the samples (1/67 for G40C and 3/67 for C147T). We also scanned all the preS2 deletion sites in our data. It was found that pre-S2 deletion mutants were present in 85 sera and 87 tumor samples, respectively. However, pre-S2 deletion mutants were not associated with prognosis of HCC patients in our data (Supplementary Figure 3).

**Table 2:** Association of the combined HBV variant with clinical variables^†^.

**Variable**	**Level**	**The presence of the two HBV variants**		
		**No**	**Yes**	***P* value**
Microscopic vascular invasion	No	50 (48.5)	15 (14.6)	8.35x10^-4^
	Yes	16 (15.5)	22 (21.4)	
Tumor size (cm)		5.89 ± 3.09	7.78 ± 3.88	0.019
GGT (U/L)		86.3 ± 99.0	148.9 ± 122.2	1.52x10^-4^
ALP (U/L)		95.0 ± 48.9	146.9± 184.5	1.15x10^-3^

†Plus/minus values are means ± SD; Data are number (%), unless otherwise indicated. Wilcoxon rank sum test was performed for continuous variables. Chi-square test was applied for count data. The two HBV variants (G40C and C147T) were combined. GGT, *γ*-glutamyltranspeptidase; ALP, alkaline phosphatase.

All virological factors including HBV genotype and HBV variants and clinicopathological factors were subjected to the Cox proportional hazard model analysis to estimate postoperative survival. Significant variables in the univariate Cox regression analysis were included in the multivariate Cox model. The results show that tumor rupture (HR=3.91, 95% CI=1.14–13.35, *p*=0.03), microscopic vascular invasion (HR=3.03, 95% CI=1.72–5.34, *p*<0.001), α-fetoprotein (AFP) (HR=1.88, 95% CI=1.04–3.38, *p*=0.036), and ALP (HR=2.5, 95% CI=1.39–4.49, *p*=0.002) increased the risk of HCC recurrence, while age (HR=0.97, 95% CI=0.94–0.99, *p*=0.024) and antiviral treatment (HR=0.15, 95% CI=0.08–0.28, *p*<0.001) significantly decreased the risk of HCC recurrence (Supplementary Table 4). Because antiviral therapy is a very strong protective factor to prevent the recurrence of HCC, the multivariate Cox proportional hazard models were further stratified by antiviral treatment. The results show that G40C (HR=3.89, 95% CI=1.39–10.87, *p*=0.01), advanced BCLC staging (HR=4.63, 95% CI=1.53–14.02, *p*=0.007), and high level of AFP (HR=5.88, 95% CI=1.88–18.39, *p*=0.002) significantly increased the risk of HCC recurrence in the group with postoperative antiviral treatment; however, G40C was not associated with postoperative recurrence in HCC patients without postoperative antiviral treatment (Supplementary Table 5).

**Figure 2:**
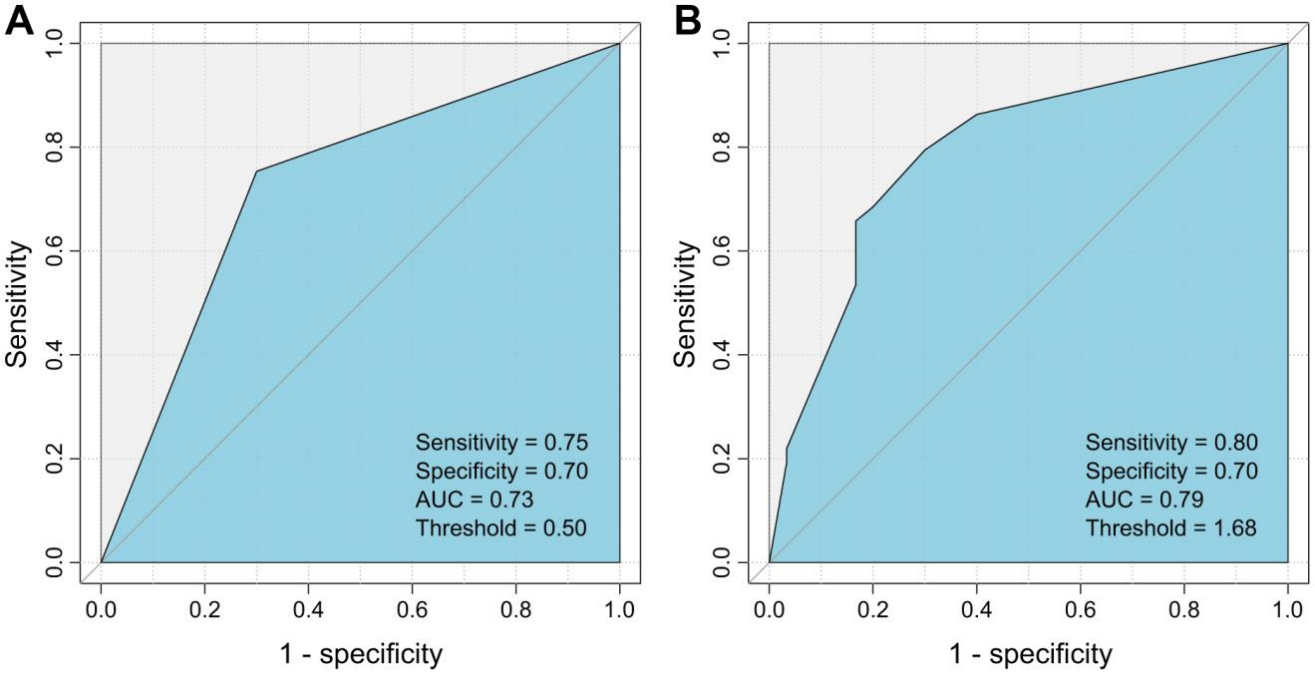
**ROC curves for the HCC recurrence prediction models using AFP alone and AFP plus G40C.** (**A**) Model using AFP alone. (**B**) Model using AFP and G40C. ROC, receiver operating characteristic; AFP, α-fetoprotein; HCC, hepatocellular carcinoma.

Next, each clinical variable and the two HBV variants were introduced into the Cox proportional hazards model to build a model that could predict postoperative recurrence of HCC. It was found that the model consisting of serum AFP and G40C achieved the best performance (area under curve (AUC) = 0.79, sensitivity= 0.80, and specificity= 0.70). In this model, AFP was further discretized to achieve a better performance (Table 3). If the model was built by dichotomized AFP (≤20 or >20 ng/mL) alone, the prediction power was less optimal (AUC= 0.73, sensitivity=0.75, specificity=0.70) (Figure 2). The statistical test suggested that the AUC of the model containing AFP and G40C was significantly larger than that of the model containing AFP alone (*p* = 0.029).

**Table 3:** Risk scores of HCC recurrence based on the presence of G40C and discretized AFP levels†.

**AFP (ng/mL)**	**Discretized AFP**	**G40C**	**Score**
<20	0	0	0
≥20 and <200	1	0	1.365
≥200 and <400	2	0	2.730
≥400	3	0	4.095
<20	0	1	2.000
≥20 and <200	1	1	3.361
≥200 and <400	2	1	4.727
≥400	3	1	6.092

†AFP, α-fetoprotein; HCC, hepatocellular carcinoma.

### Gene dysregulation and biological functions related to the two HBV variants

**Figure 3:**
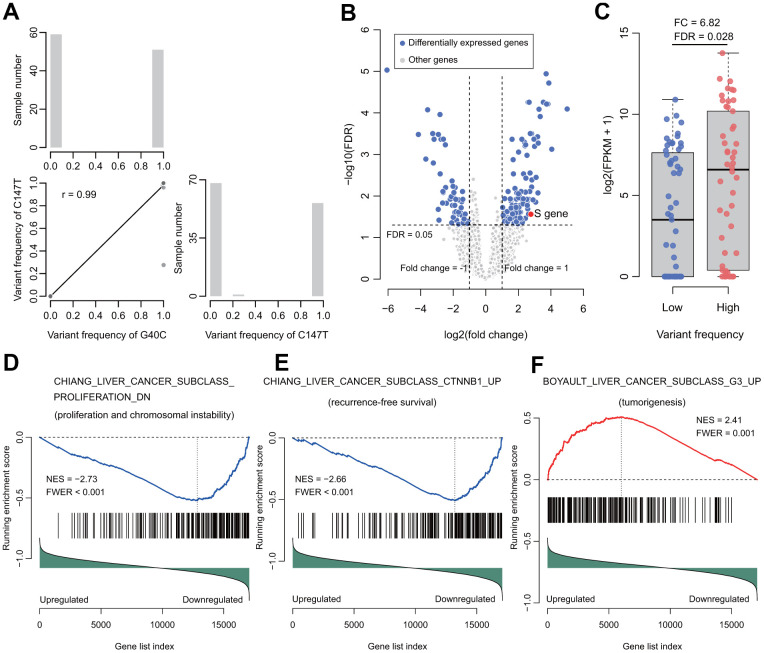
**Correlation of the two variants and their functional analysis in RNA-seq data of HBV-HCC tumors.** (**A**) The correlation (lower triangle) and frequency distributions (diagonal) of these two variants. The two variants tended to occur simultaneously. (**B**) Volcano plot for differentially expressed genes between groups with high or low frequency of the two variants. (**C**) The HBV S gene was significantly upregulated. (**D**) The gene set represents the gene signature of proliferation and chromosome instability. (**E**) The gene set represents the gene signature of recurrence-free survival. (**F**) The gene set represents the gene signature of tumorigenesis. NES, normalized enrichment score. FWER, familywise-error rate.

In the RNA-seq data, the correlation of these two HBV variants in the tumors was 0.99 (Figure 3A), which was quite consistent with the Sanger sequencing data. Furthermore, each of the variants could be identified in almost each read covering this HBV nucleotide in any positive sample. Based on this observation, the tumors in RNA-seq dataset were split into two groups: tumors with high and low frequencies of these variants. In total, 109 tumor samples with sufficient coverage on these variants were retained for differential expression analysis. It was found that 169 genes were differentially expressed between the two groups. Among these genes, 114 were upregulated, while 55 downregulated in the tumors with high frequencies of the HBV variants (Figure 3B and Supplementary Table 6). We observed that the HBV S gene was upregulated in the tumors with high frequency of the HBV variants (fold change = 6.82, FDR = 0.028, Figure 3B, 3C). G40C and C147T were located at the promoter region of the HBV S gene. Therefore, transcription factor binding sites (TFBSs) were predicted for the promoter region (nt. 1 to nt. 154 of the HBV genome). Multiple possible TFBSs were discovered at this region. These data suggest that the variants might be involved in regulating the transcription of HBV S gene (Supplementary Table 7).

Next, gene set enrichment analysis (GSEA) was performed to investigate the gene sets enriched between these two groups of tumors. In total, 47 positively and 5 negatively enriched gene sets were identified in the data (Supplementary Table 8). In the five negatively enriched gene sets, the top ranked gene set suggested that these HBV variants were associated with proliferation and chromosome instability (CHIANG_LIVER_CANCER_SUBCLASS_PROLIFERATION_DN, normalized enrichment score (NES) = -2.73, familywise-error rate (FWER) < 0.001) (Figure 3D). In addition, its complementary gene set “CHIANG_LIVER_CANCER_SUBCLASS_ PROLIFERATION_UP” was enriched positively in the data (NES = 2.62, FWER < 0.001) (Supplementary Table 8). Another top ranked negatively enriched gene set was “CHIANG_LIVER_CANCER_SUBCLASS_CTNNB1_UP” (NES = -2.66, FWER < 0.001), which confirmed the results of our survival analyses (Figure 3E). Besides these gene sets, other prognosis-predicting gene sets were significantly enriched, including LEE_LIVER_CANCER_SURVIVAL_UP (NES = -2.32, FWER = 0.005) and LEE_LIVER_CANCER_SURVIVAL_DN (NES = 2.39, FWER = 0.001), indicating that the HBV variants predicted unfavorable OS. In addition, SOTIRIOU_BREAST_CANCER_GRADE_1_VS_3_UP (NES = 2.41; FWER < 0.001) and VILLANUEVA_LIVER_CANCER_KRT19_UP (NES = 2.16, FWER = 0.01) were significantly enriched, indicating that the two HBV variants facilitate metastasis. Our results also suggest that these HBV variants are associated with tumorigenesis, anticancer drug resistance, and interferon response (BOYAULT_LIVER_CANCER_SUBCLASS_G3_UP, NES = 2.41, FWER = 0.001; KOBAYASHI_EGFR_SIGNALING_24HR_DN, NES = 2.32, FWER = 0.001; and FARMER_BREAST_CANCER_CLUSTER_1, NES = -2.15, FWER = 0.051) (Figure 3F and Supplementary Table 8).

## DISCUSSION

In this study, RNA-seq datasets of 203 HCC samples were firstly screened for HCC prognosis-related HBV variants in the preS region. A total of 12 variants related to RFS were initially identified. Of those, G40C and C147T were successfully validated both in the sera and in the tumors of 103 HBV-HCC patients in our prospective cohort. Interestingly, the two variants were actually polymorphic sites between the genomes of HBV genotypes B2 and C2 and highly linked with each other, either in the RNA-seq data of the tumor tissues or in the Sanger sequencing data of both the sera and the tumor tissues. G40C is also a representative of HBV genotype B2 or genotype mixture with genotype B2. Compared to genotype C2, HBV genotype B2 or genotype mixture increases the risk of HCC recurrence, which is concordant with our previous study [15]. The results of multivariate Cox proportional hazard models show that G40C, advanced BCLC staging, and high level of AFP significantly increased the risk of HCC recurrence in the group with postoperative antiviral treatment, while there was no effect in the group without postoperative antiviral treatment (Supplementary Table 5). Antiviral treatment can decrease the occurrence and recurrence of HCC in high-risk HBV-infected subjects [14, 16, 17]. This result indicates that postoperative antiviral treatment could not decrease the risk of HCC recurrence in patients with HBV genotype B carrying G40C.

Our results show that G40C as a represent of C147T and HBV genotype B2 was a significant serological biomarker for HCC recurrence. AUC of the optimized model consisting of HBV G40C and AFP in the sera was 0.79. The prediction power of this prediction model is better than the one we previously developed using clinical variables composed of the levels of HBV DNA load, the presence of liver cirrhosis, the level of AFP, and BCLC stage [18]. Furthermore, the Cox prediction model based on the AJCC tumor stage and ratios of serum preS2 deletion as deleted by a preS gene chip was also developed for the prediction of postoperative prognosis in HBV-HCC [12, 19]. It has been confirmed that the AUC of this model is 0.741 in the main cohort and 0.704 in the validation cohort [12]. Apparently, our model established in this study should be more powerful than the currently published ones in predicting postoperative prognosis in HBV-HCC. This model is worth translating into clinical practice.

Some studies have confirmed the carcinogenic potential of the preS2 mutated proteins in both transgenic mice and cell culture [7, 20, 21]. The rtM204I/sW196* preS/S truncation induce the cell transformation and tumorigenesis ability via altered host gene expressions, including MGST2, HIF1A, and TGFbi. Downregulated TGFbi may be a common mechanism for oncogenicity in HBV surface truncation mutants [22]. PreS2 deletions modulate cellular processes with a potential impact on liver disease. The accumulation of mutated envelope proteins in the ER leads to ER stress, DNA damage, centrosome overduplication, and genomic instability [23–25]. HBV preS2 interacted with the preS2-responsible region and activated the hTERT promoter, resulting in the upregulation of telomerase activity and the promotion of HCC development [22]. However, the mechanism by which the nucleotide variants in the preS2 of HBV promote the recurrence of HCC remains unknown. G40C and C147T are located in the HBV preS2 region and as well as the promoter region of the HBV S gene. The expression of the HBV S gene was upregulated 6.82 times in the tumors with high frequency of the two variants, compared to those with low frequency of the two variants (Figure 3C). The HBV variants may alter the binding of the TFBSs to the promoter, which regulates the transcription of HBV S gene (Supplementary Table 7). In this study, we also provided evidence showing that the two HBV variants facilitated cell proliferation, chromosome instability, tumorigenesis, metastasis, and anticancer drug resistance. It may explain the reason that HBV Genotype B2 increases the risk of HCC recurrence. Further experimental studies using cell lines and animal models are suggested to validate the cancer promoting function of the HBV with the two variants.

In summary, the present study indicates that HBV preS variants G40C and C147T as representatives of HBV Genotype B2 are highly linked with each other, and may serve as prognostic biomarkers in both sera and tumor tissue samples of HBV-HCC patients. The AUC of the optimized model combining G40C with AFP was 0.79. HBV preS G40C variant and serological AFP are easily examined in HBV-HCC patients and helpful for making therapeutic decision before surgery. Thus, the model is worth translating into clinical practice.

## MATERIALS AND METHODS

### RNA-seq data analysis to screen for HBV variants related to HCC prognosis

The original RNA-seq data of 203 HCC patients from 12 studies (SRP062885, SRP069212, SRP099053, SRP174991, SRP256409, SRA074279, SRP039694, SRP108560, SRP118972, SRP120360, SRP188371, and SRP220071) were retrieved from the Sequence Read Archive database [26–34]. HBV variants in the region of preS1/preS2 were extracted as previously described and those with average frequencies ≥ 25% and more than 100 valid values were kept for downstream analyses [35]. The read count matrix was obtained by Salmon [36]. During the process, the annotation of human genes was combined with that of HBV ones. Thus, the abundances of HBV genes were evaluated along with the human genes during the quantification process. Combat-Seq method was applied to adjust the potential batch effect among different studies [37]. The gene set names containing “survival” (or “recurrence”) and “liver cancer” were retrieved from gene sets of chemical and genetic perturbations in MsigDB (http://software.broadinstitute.org/gsea/index.jsp) as liver-specific prognosis-related gene sets. Sample-level prognosis scores were calculated by gene set variation analysis in which classical maximum deviation method was performed to compute the enrichment statistics [38]. The parameter “min.sz” was set to 10 during the process. The function “cor.test” in R language was applied to calculate the Pearson correlations between variant frequencies of HBV loci and prognosis scores. The Benjamini–Hochberg (BH) method was performed among the prognostic gene sets per variant to calculate FDRs. Any association with FDR < 0.1 was kept for downstream analyses. For differential expression gene analysis, edgeR was applied [39, 40]. *P* values were adjusted by BH method. Genes with fold change ≥ 2 and FDR < 0.05 were collected as differentially expressed genes. For GSEA analysis, the gene’s read count was converted into Fragments Per Kilobase of exon model per Million mapped fragments (FPKM). Taking the signal-to-noise ratio as input, the “GSEAPreranked” tool in GSEA software was performed to detect the gene sets enriched in the data [41]. Gene sets with FWER ≤ 0.1 were considered as significantly enriched.

### Independent validation of the HBV variants in HBV-HCC patients

In total, 103 consecutive HBV-infected HCC patients who received radical hepatectomy from this research group of the Eastern Hepatobiliary Surgery Hospital (Shanghai, China) were enrolled and confirmed by pathology from February 2011 to March 2012. Resected tumors were subjected to pathological examination for tumor-free resection margin > 1 cm without evidence of cancer metastasis. Preoperative peripheral blood samples and tumor tissues of participants were collected and stored at -80° C immediately after surgery. Routine laboratory tests related to liver function were measured using international standard methods and matched reagents (HITACHI 7600, Hitachi Koki Co. Ltd., Hitachinaka City, Japan; Wako Diagnostics Reagents, Wako Pure Chemical Industries Ltd., Osaka, Japan). Alpha-fetoprotein concentrations were routinely measured on the Cobas e601 immunoassay analyzers and matched reagents (Roche Diagnostics, Manheim, Germany) with electrochemiluminescence technology. Participants were surgically treated and followed-up according to the standard protocols as previously described [14]. The follow-up was finished on October 1^st^, 2019. All participants were self-reported Han Chinese. This study was approved by the ethics committee of Eastern Hepatobiliary Surgery Hospital. All patients provided written informed consent.

HBV DNA of preoperative sera and tumors was extracted using QIAamp DNA blood mini kit (Qiagen, Hilden, Germany). The HBV genome between nt.2743 and nt.255 (from nt.2743 to nt.3215 and from nt.1 to nt.255) was amplified using nested PCR and sequenced using the cloning-based sequencing method as previously described [42]. Ten clones of each sample were randomly selected for Sanger sequencing. Genotyping was performed by HBV subtype analyzer (STAR) as previously described [35]. For a clone, scores were assigned for Genotype A to H. The genotype of a clone was identified as the one with the largest score. Samples with clones of multiple genotypes were defined as mixture. Variants of each clone and preS2 deletion sites were retrieved from BLAST alignments [43]. Clones that failed to align to the HBV genome were excluded from the subsequent analysis. Sample-level variants were then summarized via collecting the variants of all clones in a sample. In simple terms, in any clone of a sample, if a variant was detected at a nucleotide of the HBV genome, then we considered that the variant was present at that locus in that sample. The pairwise distances of the clones from serum samples were calculated by MEGA X and then visualized to inspect identical clones and therefore inter-subject contamination [44].

### Statistical analysis

Clinical and baseline characteristics were summarized by using mean values with standard deviation or median values with interquartile range (IQR, 25th to 75th percentiles) for continuous variables. Proportions were applied for categorical values. Univariate Cox regression analysis and log-rank test were applied to estimate the associations between the presence of the viral variants and patients’ OS and RFS. Kaplan–Meier method performed survival analysis and generated a survival plot. The selected HBV variants and each clinical variable were subjected to the Cox regression analyses to compute the risk scores and build HCC recurrence prediction models. Given that *x_i_* is the *i*th variable and *β_i_* is its coefficient, then the risk score of a patient is calculated as:

$$Risk\;Score=\;\sum_{i=1}^{n}\beta_{i}\;\chi_{i}$$

The number of variables (*i.e.*, *n* in the formula) was set to 2 according to the Harrell’s guidelines [45]. The best one was determined by the AUC. The receiver operating characteristic (ROC) curves were plotted by R package pROC [46]. The statistical significance between two AUCs was determined by the one-sided test applied by the function “roc.test” in the pROC package with default parameters. TFBS were predicted by Find Individual Motif Occurrences [47]. The match *p*-value was set to 0.001 to gain more sensitivity. TFBSs with false discovery rate (FDR) of < 0.25 and overlap with G40C or C147T were collected. For clinical variable association test, differences were determined by Wilcoxon rank sum test or χ^2^ tests as appropriate. P<0.05 was considered significant. All analyses were two-side and performed using SPSS, version 21 (Armonk, NY).

### Ethics committee approval

The study protocol conformed to the ethical guidelines of the 1975 Declaration of Helsinki and was approved by the ethics committee of Eastern Hepatobiliary Surgery Hospital. All patients provided written informed consent.

### Availability of data and materials

The sequences of clone sequencing were deposited in GenBank with accession numbers MW179612 - MW180878.

## Supplementary Material

Supplementary Figures

Supplementary Tables 1-5

Supplementary Table 6

Supplementary Tables 7, 8
