# Supplementary material for: Nucleotide variants in hepatitis B virus preS region predict the recurrence of hepatocellular carcinoma
Source: Aging (Albany NY). 2021 Sep 17;13(18):22256–75. doi: 10.18632/aging.203531 (PMC8507287; doi:10.18632/aging.203531)
Supplement: Supplementary Figures [file aging-13-203531-s001.pdf]

SUPPLEMENTARY FIGURES

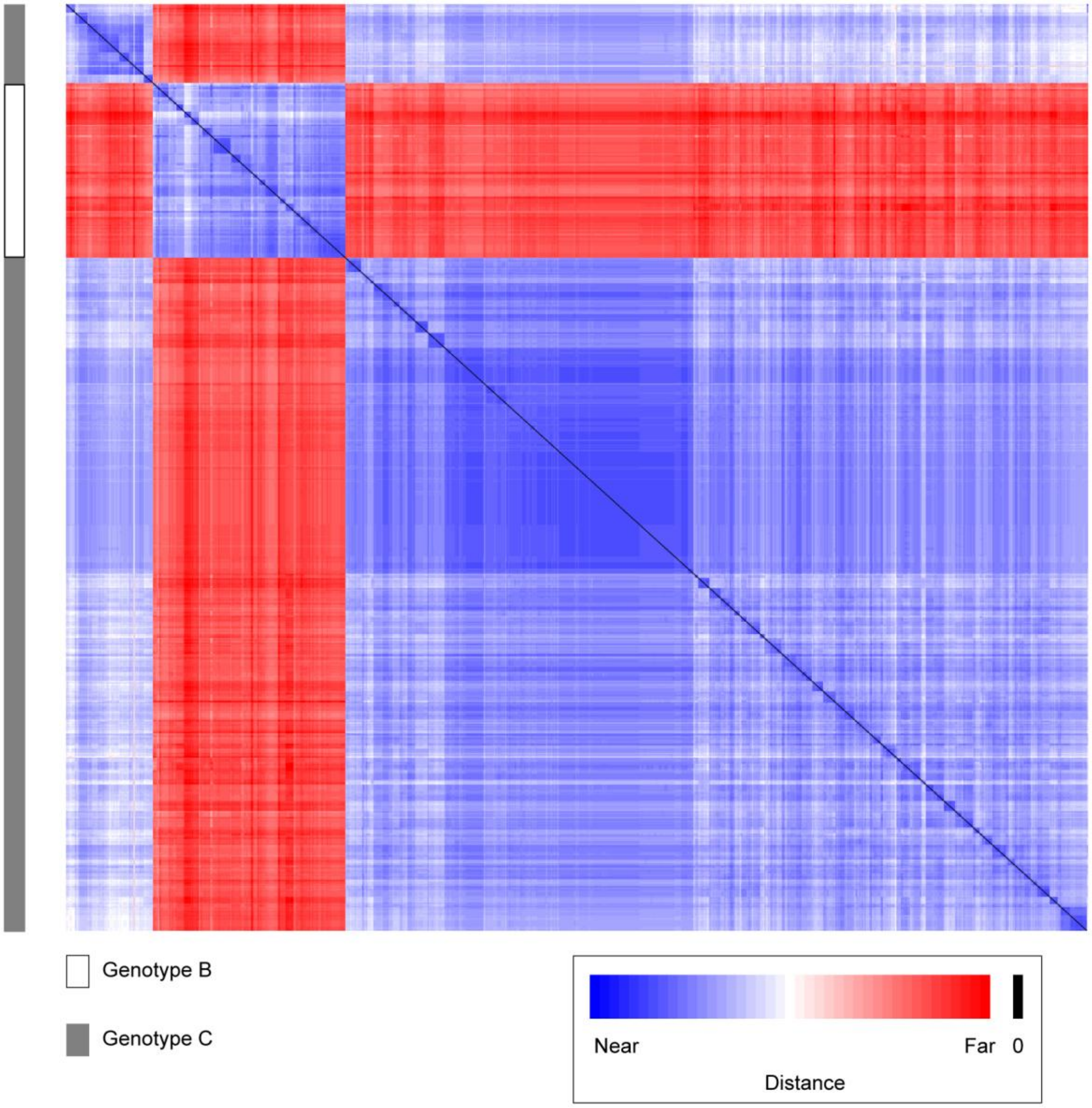

Supplementary Figure 1. Heat map of pairwise distances of the clones from the serum samples. Every row or column represents a clone. The grids on the diagonal line represent the distances between a clone and itself, which are all 0 (marked by black).

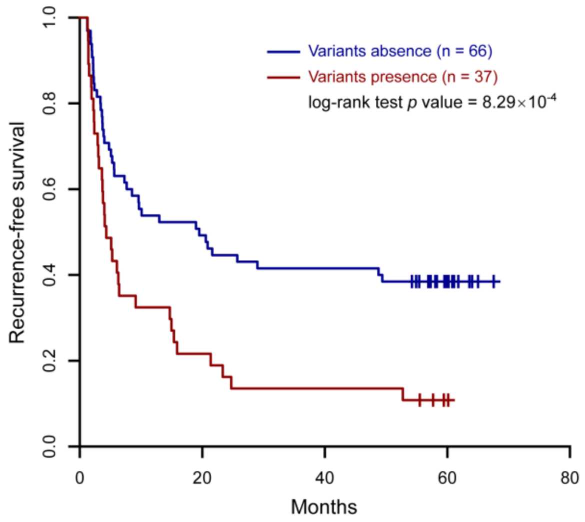

Supplementary Figure 2. Combined variants predicted unfavorable recurrence-free survival. G40C and C147T were combined because of their high frequency of concurrence. Kaplan–Meier curve was plotted to visualize the prognosis difference.

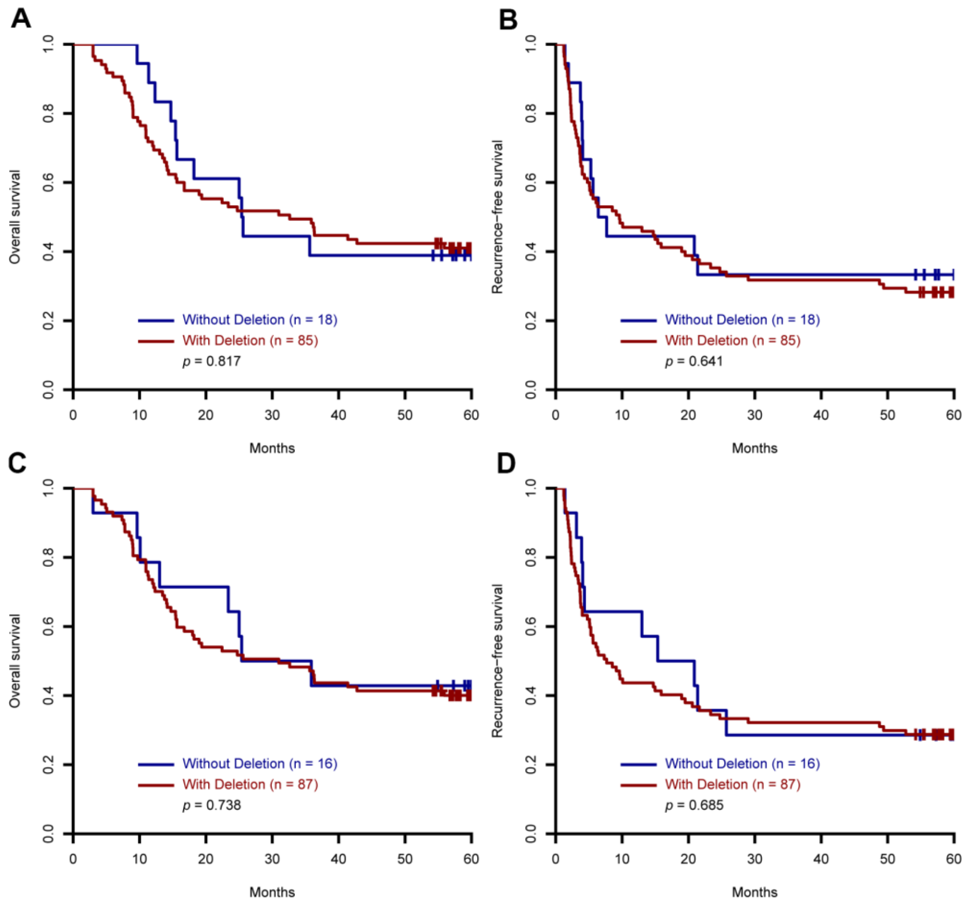

Supplementary Figure 3. The prognostic value of the preS2-deletion mutations in the HCC patients from our cohort. (**A**) the serum samples, overall survival; (**B**) the serum samples, recurrence-free survival; (**C**) tumoral samples, overall survival; (**D**) tumoral samples, recurrence-free survival.
