# Supplementary material for: Nucleotide variants in hepatitis B virus preS region predict the recurrence of hepatocellular carcinoma
Source: Aging (Albany NY). 2021 Sep 17;13(18):22256–75. doi: 10.18632/aging.203531 (PMC8507287; doi:10.18632/aging.203531)
Supplement: Supplementary Tables 1-5 [file aging-13-203531-s002.pdf]

SUPPLEMENTARY TABLES

Supplementary Table 1. Liver cancer-specific and prognosis-related gene sets.

Gene set	Pubmed ID
**Overall survival-related gene sets**	
LEE_LIVER_CANCER_SURVIVAL_DN	15349906
LEE_LIVER_CANCER_SURVIVAL_UP	15349906
HOSHIDA_LIVER_CANCER_SURVIVAL_UP	18701503
HOSHIDA_LIVER_CANCER_SURVIVAL_DN	18701503
KIM_LIVER_CANCER_POOR_SURVIVAL_UP	21320499
KIM_LIVER_CANCER_POOR_SURVIVAL_DN	21320499
**Recurrence-free survival-related gene sets**	
IIZUKA_LIVER_CANCER_EARLY_RECURRENCE	12648972
HOSHIDA_LIVER_CANCER_LATE_RECURRENCE_UP	18923165
HOSHIDA_LIVER_CANCER_LATE_RECURRENCE_DN	18923165
WOO_LIVER_CANCER_RECURRENCE_UP	18381945
WOO_LIVER_CANCER_RECURRENCE_DN	18381945
KUROKAWA_LIVER_CANCER_EARLY_RECURRENCE_UP	15288478
KUROKAWA_LIVER_CANCER_EARLY_RECURRENCE_DN	15288478

Supplementary Table 2. Association between HBV variants frequencies and HCC recurrence-related gene sets.

Variant	Gene set	Pearson correlation	*P* value	FDR
G40C	KUROKAWA_LIVER_CANCER_EARLY_RECURRENCE_UP	0.29	2.11E-03	2.54E-02
G45C	KUROKAWA_LIVER_CANCER_EARLY_RECURRENCE_UP	0.28	2.19E-03	2.63E-02
A85G	KUROKAWA_LIVER_CANCER_EARLY_RECURRENCE_UP	0.27	2.21E-03	2.65E-02
A87G	KUROKAWA_LIVER_CANCER_EARLY_RECURRENCE_UP	0.25	5.39E-03	6.47E-02
T93C	KUROKAWA_LIVER_CANCER_EARLY_RECURRENCE_UP	0.26	4.51E-03	5.42E-02
C96A	KUROKAWA_LIVER_CANCER_EARLY_RECURRENCE_UP	0.25	4.77E-03	5.72E-02
C99A	KUROKAWA_LIVER_CANCER_EARLY_RECURRENCE_UP	0.26	4.13E-03	4.96E-02
C105T	KUROKAWA_LIVER_CANCER_EARLY_RECURRENCE_UP	0.26	3.91E-03	4.69E-02
C110G	KUROKAWA_LIVER_CANCER_EARLY_RECURRENCE_UP	0.25	6.11E-03	7.33E-02
C127A	KUROKAWA_LIVER_CANCER_EARLY_RECURRENCE_UP	0.26	3.69E-03	4.43E-02
G132A	KUROKAWA_LIVER_CANCER_EARLY_RECURRENCE_UP	0.28	1.82E-03	2.19E-02
C147T	KUROKAWA_LIVER_CANCER_EARLY_RECURRENCE_UP	0.24	6.87E-03	8.24E-02

Abbreviations: FDR, false discovery rate.

Supplementary Table 3. Recurrence-related HBV variants validated by sanger sequencing in the tumors.

Variant	Hazard ratio	95% confidence interval	*P* value
G40C	1.78	1.04–3.05	4.49E-02
G45C	1.63	0.95–2.79	8.63E-02
A85G	1.74	1.02–2.98	5.30E-02
A87G	1.72	1.01–2.91	5.45E-02
T93C	1.54	0.90–2.62	1.29E-01
C96A	1.74	1.02–2.98	5.30E-02
C99A	1.60	0.95–2.71	9.00E-02
C105T	0.94	0.55–1.61	8.31E-01
C110G	1.69	0.98–2.92	7.23E-02
C127A	1.43	0.86–2.40	1.83E-01
G132A	1.59	0.95–2.67	8.94E-02
C147T	1.74	1.03–2.95	4.84E-02

Supplementary Table 4. Cox regression analysis for the factors significantly affected the recurrence of postoperative HCC patients.

Variables		No. (%) of patients (n=103)	Univariate analysis		Multivariate analysis	
			HR (95% CI)	*P* value	HR (95% CI)	*P* value
Age (years)		103 (100)	0.96 (0.94–0.99)	0.008	0.97 (0.94–0.99)	0.024
HBV genotype	Genotype C	67 (65.05)	1			
	Not Genotype C	9 (8.74)	1.97 (1.23–3.15)	0.005		
G40C	G	66 (64.1)	1			
	C	37 (35.9)	2.17 (1.36–3.47)	0.001		
C147T	C	64 (62.1)	1			
	T	39 (37.9)	1.83 (1.15–2.92)	0.01		
Tumor rupture	No	100 (97.1)	1		1	
	Yes	3 (2.9)	4.57 (1.40–14.91)	0.012	3.91 (1.14–13.35)	0.03
Portal vein tumor thrombi	No	84 (81.6)	1			
	Yes	19 (18.4)	4.24 (2.44–7.36)	<0.001		
Tumor size	<3cm	16 (15.5)	1			
	≥3cm	87 (84.5)	2.52 (1.16–5.52)	0.02		
Tumor capsule	Complete	16 (15.5)	1			
	Incomplete/Absence	87 (84.5)	2.55 (1.17–5.57)	0.019		
Microscopic vascular invasion	No	65 (63.1)	1		1	
	Yes	38 (36.9)	5.26 (3.22–8.59)	<0.001	3.03 (1.72–5.34)	<0.001
Tumor differentiation	I/II	21 (20.4)	1			
	III	82 (79.6)	2.82 (1.40–5.69)	0.004		
BCLC staging	0/A	36 (35.0)	1			
	B/C	67 (65.0)	3.00 (1.74–5.20)	<0.001		
Postoperative antiviral treatment	No	58 (56.3)	1		1	
	Yes	45 (43.7)	0.17 (0.10–0.29)	<0.001	0.15 (0.08–0.28)	<0.001
AFP	≤20 ng/mL	42 (40.8)	1		1	
	>20 ng/mL	61 (59.2)	2.78 (1.62–4.76)	<0.001	1.88 (1.04–3.38)	0.036
AST (U/L)	≤37	48 (46.6)	1			
	>37	55 (53.4)	1.71 (1.07–2.74)	0.024		
GGT (U/L)	≤61	50 (48.5)	1			
	>61	53 (51.5)	1.99 (1.25–3.18)	0.004		
ALP (U/L)	≤129	84 (81.6)	1		1	
	>129	19 (18.4)	2.05 (1.19–3.54)	0.01	2.50 (1.39–4.49))	0.002

Abbreviations: CI, confidence interval; HBV, hepatitis B virus; HCC, hepatocellular carcinoma; HR, hazard ratio; AST, aspartate aminotransferase; GGT, γ –glutamyltranspeptidase; AFP, α-fetoprotein; ALP, alkaline phosphatase.

Supplementary Table 5. Cox regression analysis for the factors significantly affected the recurrence of postoperative HCC patients stratified by antiviral treatment.

**Variables**		**Multivariate analysis**	
		**HR (95% CI)**	***P* value**
**With postoperative antiviral treatment (n=45)**			
G40C	G	1	
	C	3.89 (1.39–10.87)	0.01
BCLC staging	0/A	1	
	B/C	4.63 (1.53–14.02)	0.007
AFP (ng/mL)	≤20	1	
	>20	5.88 (1.88–18.39)	0.002
**Without postoperative antiviral treatment (n=58)**			
Age (years)		0.96 (0.93–0.99)	0.024
Tumor rupture	No	1	
	Yes	5.07 (1.43–17.99)	0.012
Microscopic vascular invasion	No	1	
	Yes	4.00 (2.07–7.72)	<0.001
ALP (U/L)	≤129	1	
	>129	2.85 (1.43–5.65)	0.003

Abbreviations: CI, confidence interval; HCC, hepatocellular carcinoma; HR, hazard ratio; AFP, α-fetoprotein; ALP, alkaline phosphatase.
