# Supplementary material for: Nucleotide variants in hepatitis B virus preS region predict the recurrence of hepatocellular carcinoma
Source: Aging (Albany NY). 2021 Sep 17;13(18):22256–75. doi: 10.18632/aging.203531 (PMC8507287; doi:10.18632/aging.203531)
Supplement: Supplementary Table 6 [file aging-13-203531-s003.docx]

**Supplementary Table 6. Differentially expressed genes in the tumors with high/low frequencies of two HBV variants.**

| **Gene** | **log2(fold change)** | **log2(CPM)** | ***P* value** | **FDR** |
| --- | --- | --- | --- | --- |
| RN7SL3 | -6.06 | 5.38 | 7.13E-10 | 9.33E-06 |
| CPNE6 | -4.14 | 1.90 | 2.39E-07 | 3.31E-04 |
| SYT8 | -3.68 | 3.25 | 1.78E-06 | 1.29E-03 |
| LINC01203 | -3.57 | -0.14 | 3.87E-08 | 8.43E-05 |
| LINC02160 | -3.26 | -1.50 | 1.99E-07 | 3.15E-04 |
| SPTA1 | -3.22 | 0.88 | 2.33E-06 | 1.61E-03 |
| AC093866.1 | -3.17 | 0.80 | 4.29E-07 | 4.31E-04 |
| IGHV2-26 | -2.92 | 1.11 | 9.14E-05 | 2.10E-02 |
| AL591686.1 | -2.91 | -0.04 | 2.40E-07 | 3.31E-04 |
| PNMA5 | -2.87 | 2.02 | 2.72E-04 | 3.81E-02 |
| AC090948.2 | -2.81 | 0.84 | 5.44E-08 | 1.09E-04 |
| RAB44 | -2.80 | -0.57 | 4.62E-06 | 2.93E-03 |
| TNNI2 | -2.75 | 1.68 | 4.04E-07 | 4.31E-04 |
| PLP1 | -2.65 | -1.77 | 5.53E-05 | 1.47E-02 |
| AL137018.1 | -2.63 | -1.07 | 1.73E-05 | 7.79E-03 |
| RPL29P11 | -2.62 | 0.45 | 1.92E-05 | 8.25E-03 |
| AL359237.1 | -2.60 | -0.75 | 3.52E-07 | 4.29E-04 |
| LINC02204 | -2.48 | -1.72 | 6.72E-07 | 5.86E-04 |
| PNLDC1 | -2.47 | -0.84 | 1.22E-05 | 6.24E-03 |
| OR51B5 | -2.42 | -0.50 | 5.66E-05 | 1.49E-02 |
| B3GNT6 | -2.18 | -0.61 | 3.77E-05 | 1.19E-02 |
| WDR63 | -2.12 | -1.79 | 2.86E-05 | 1.04E-02 |
| GABRG1 | -2.09 | -1.02 | 3.58E-04 | 4.42E-02 |
| PGM5P2 | -2.07 | 0.33 | 7.81E-06 | 4.35E-03 |
| C2-AS1 | -2.05 | -2.09 | 6.74E-05 | 1.68E-02 |
| CHP2 | -2.01 | 1.59 | 1.07E-05 | 5.85E-03 |
| KCNH1 | -2.01 | -0.12 | 2.84E-05 | 1.04E-02 |
| GDNF | -1.99 | 1.80 | 1.88E-04 | 3.25E-02 |
| SLC30A2 | -1.96 | 2.21 | 1.92E-05 | 8.25E-03 |
| HSD3B1 | -1.91 | 0.55 | 1.24E-04 | 2.46E-02 |
| AC007402.1 | -1.90 | -2.70 | 6.75E-05 | 1.68E-02 |
| LINC01931 | -1.88 | -3.00 | 1.21E-05 | 6.24E-03 |
| AC087633.1 | -1.84 | -2.43 | 3.95E-05 | 1.20E-02 |
| AL008638.3 | -1.84 | -0.97 | 1.13E-04 | 2.39E-02 |
| PROK1 | -1.79 | -1.56 | 2.76E-04 | 3.82E-02 |
| AC012085.2 | -1.68 | -2.34 | 4.06E-04 | 4.79E-02 |
| LINC01483 | -1.67 | -1.98 | 4.26E-05 | 1.24E-02 |
| AL357513.1 | -1.67 | -2.75 | 1.18E-04 | 2.45E-02 |
| LINC01727 | -1.65 | 0.80 | 1.50E-04 | 2.75E-02 |
| FO681548.1 | -1.55 | -2.68 | 1.29E-04 | 2.52E-02 |
| NECTIN3-AS1 | -1.51 | -0.16 | 8.12E-05 | 1.91E-02 |
| MIR4300HG | -1.51 | -2.54 | 3.98E-04 | 4.71E-02 |
| AC044839.1 | -1.48 | -0.33 | 4.98E-05 | 1.37E-02 |
| CR381670.2 | -1.46 | 1.14 | 1.65E-04 | 2.92E-02 |
| RYR3 | -1.43 | 1.55 | 1.71E-05 | 7.79E-03 |
| LRRTM4 | -1.41 | 1.13 | 4.04E-05 | 1.20E-02 |
| AC009242.1 | -1.39 | -1.26 | 3.61E-04 | 4.44E-02 |
| EDA2R | -1.37 | 1.15 | 2.74E-04 | 3.82E-02 |
| SLC4A1 | -1.33 | 0.53 | 2.39E-04 | 3.57E-02 |
| TMIE | -1.26 | 0.90 | 1.47E-04 | 2.72E-02 |
| MDGA1 | -1.25 | 2.42 | 3.22E-04 | 4.14E-02 |
| FIRRE | -1.18 | 1.88 | 3.51E-04 | 4.39E-02 |
| RBM20 | -1.11 | 1.43 | 3.88E-04 | 4.63E-02 |
| DNMBP-AS1 | -1.09 | 0.29 | 3.10E-04 | 4.05E-02 |
| AC016995.1 | -1.05 | 1.62 | 2.96E-04 | 3.95E-02 |
| LZTS1 | 1.04 | 2.57 | 7.94E-05 | 1.89E-02 |
| FIGNL2 | 1.07 | 3.03 | 1.64E-04 | 2.92E-02 |
| ZNF503-AS1 | 1.16 | -1.01 | 3.31E-05 | 1.16E-02 |
| COL9A1 | 1.16 | 0.02 | 3.11E-04 | 4.05E-02 |
| MGAT3 | 1.21 | 0.93 | 1.29E-04 | 2.52E-02 |
| PTK7 | 1.22 | 3.90 | 1.99E-04 | 3.31E-02 |
| CACNB4 | 1.25 | 1.66 | 2.00E-04 | 3.31E-02 |
| ITM2A | 1.25 | 3.64 | 2.38E-04 | 3.57E-02 |
| ZNF853 | 1.28 | 1.13 | 1.23E-04 | 2.46E-02 |
| TRGC1 | 1.30 | 0.50 | 3.84E-04 | 4.61E-02 |
| SLC28A2 | 1.30 | 2.60 | 2.58E-04 | 3.68E-02 |
| AUXG01000058.1 | 1.31 | 1.55 | 1.09E-04 | 2.37E-02 |
| CACNA2D2 | 1.34 | 0.49 | 1.91E-04 | 3.25E-02 |
| NPAS1 | 1.35 | -0.04 | 3.23E-04 | 4.14E-02 |
| ERVE-1 | 1.37 | 1.07 | 1.65E-05 | 7.79E-03 |
| STAR | 1.41 | 0.56 | 1.77E-04 | 3.11E-02 |
| TMEM145 | 1.49 | 1.44 | 2.35E-04 | 3.55E-02 |
| P2RY14 | 1.51 | 1.15 | 3.58E-05 | 1.18E-02 |
| SCN2B | 1.52 | -1.93 | 2.29E-04 | 3.52E-02 |
| ANXA3 | 1.52 | 1.79 | 1.49E-04 | 2.74E-02 |
| NCAM1 | 1.54 | 1.47 | 1.12E-04 | 2.39E-02 |
| ANKRD20A19P | 1.55 | -0.23 | 4.64E-05 | 1.31E-02 |
| XIRP1 | 1.55 | -1.90 | 1.99E-04 | 3.31E-02 |
| UNC79 | 1.55 | -0.36 | 3.59E-05 | 1.18E-02 |
| TRIM67 | 1.56 | 0.20 | 8.33E-05 | 1.95E-02 |
| EIF4BP6 | 1.57 | -1.62 | 2.24E-04 | 3.49E-02 |
| MEST | 1.61 | 6.14 | 4.29E-07 | 4.31E-04 |
| KCNC1 | 1.62 | -0.43 | 6.67E-05 | 1.68E-02 |
| TMEM125 | 1.64 | -0.52 | 1.42E-04 | 2.70E-02 |
| CHRNB2 | 1.65 | -0.79 | 1.02E-04 | 2.26E-02 |
| MAP1A | 1.68 | 2.58 | 1.89E-06 | 1.34E-03 |
| GSTP1 | 1.70 | 5.21 | 4.56E-06 | 2.93E-03 |
| DDX11L10 | 1.71 | -1.64 | 1.31E-04 | 2.54E-02 |
| AHNAK2 | 1.74 | 2.83 | 3.70E-04 | 4.50E-02 |
| COL9A2 | 1.79 | 3.09 | 1.31E-05 | 6.58E-03 |
| POU3F1 | 1.82 | -1.17 | 1.92E-04 | 3.25E-02 |
| SPTSSB | 1.83 | 1.03 | 2.06E-04 | 3.38E-02 |
| AC022167.4 | 1.83 | -2.41 | 1.33E-04 | 2.54E-02 |
| MAGEA10 | 1.85 | 0.54 | 6.81E-05 | 1.68E-02 |
| TTYH1 | 1.86 | 2.06 | 7.81E-05 | 1.87E-02 |
| VAT1L | 1.88 | 2.18 | 9.44E-05 | 2.13E-02 |
| ZFHX4-AS1 | 1.88 | 0.35 | 3.22E-04 | 4.14E-02 |
| NPW | 1.89 | 3.44 | 3.74E-05 | 1.19E-02 |
| COL11A2 | 1.89 | 1.78 | 2.13E-05 | 8.44E-03 |
| RPL10P6 | 1.90 | -1.05 | 1.93E-04 | 3.25E-02 |
| CUZD1 | 1.91 | -0.48 | 2.06E-05 | 8.44E-03 |
| AC005515.1 | 1.91 | -1.17 | 2.32E-04 | 3.54E-02 |
| REXO1L1P | 1.92 | -1.62 | 2.47E-04 | 3.59E-02 |
| SHOX2 | 1.97 | 0.84 | 3.97E-04 | 4.71E-02 |
| COL22A1 | 1.97 | 2.31 | 2.52E-04 | 3.64E-02 |
| SLC7A4 | 1.97 | -1.23 | 2.43E-04 | 3.57E-02 |
| PLD5 | 1.99 | -0.70 | 1.32E-04 | 2.54E-02 |
| GRIK1 | 2.01 | -0.78 | 2.26E-05 | 8.83E-03 |
| AL845472.1 | 2.01 | 0.08 | 1.14E-06 | 8.77E-04 |
| AIRE | 2.02 | -1.46 | 3.06E-05 | 1.10E-02 |
| ST8SIA3 | 2.02 | 0.82 | 2.20E-04 | 3.44E-02 |
| PKP3 | 2.03 | 0.45 | 2.19E-04 | 3.44E-02 |
| LEFTY2 | 2.04 | -1.74 | 7.67E-05 | 1.86E-02 |
| SLC17A8 | 2.06 | -0.63 | 1.20E-04 | 2.46E-02 |
| RN7SL145P | 2.06 | -1.78 | 2.52E-05 | 9.56E-03 |
| MAGEL2 | 2.09 | -1.97 | 2.09E-05 | 8.44E-03 |
| KCNH2 | 2.11 | 1.66 | 2.08E-04 | 3.38E-02 |
| C1QTNF3 | 2.11 | 5.66 | 6.91E-05 | 1.69E-02 |
| ARL14 | 2.13 | 0.39 | 1.18E-04 | 2.45E-02 |
| C1QTNF9B | 2.17 | -2.17 | 1.67E-05 | 7.79E-03 |
| RAB25 | 2.17 | -0.21 | 2.44E-04 | 3.57E-02 |
| NEXMIF | 2.18 | -0.57 | 2.65E-04 | 3.77E-02 |
| RFLNA | 2.19 | 0.66 | 3.60E-05 | 1.18E-02 |
| SLC22A17 | 2.22 | 2.50 | 3.99E-07 | 4.31E-04 |
| OPRPN | 2.25 | 0.81 | 3.36E-04 | 4.27E-02 |
| KRT17 | 2.29 | 1.81 | 5.88E-05 | 1.54E-02 |
| HSD3B2 | 2.31 | 0.20 | 3.44E-05 | 1.17E-02 |
| LIN28A | 2.32 | 0.94 | 4.71E-06 | 2.93E-03 |
| PLAAT5 | 2.33 | -1.00 | 3.45E-05 | 1.17E-02 |
| CYP2W1 | 2.37 | 0.36 | 5.24E-07 | 5.07E-04 |
| SEZ6L | 2.41 | -1.25 | 7.33E-07 | 6.19E-04 |
| LHFPL4 | 2.44 | 0.88 | 2.19E-04 | 3.44E-02 |
| ERP27 | 2.44 | 2.97 | 1.82E-05 | 8.07E-03 |
| DPYSL5 | 2.45 | -1.43 | 6.64E-07 | 5.86E-04 |
| CLDN6 | 2.46 | -0.78 | 6.25E-06 | 3.72E-03 |
| SLITRK2 | 2.52 | -0.58 | 4.04E-05 | 1.20E-02 |
| IGLON5 | 2.53 | 0.77 | 1.40E-06 | 1.05E-03 |
| ANXA8 | 2.53 | 0.76 | 4.96E-05 | 1.37E-02 |
| CLDN18 | 2.55 | 1.36 | 6.69E-05 | 1.68E-02 |
| BCAN | 2.58 | 2.10 | 8.80E-05 | 2.04E-02 |
| APCDD1 | 2.60 | 3.39 | 1.48E-08 | 5.55E-05 |
| CNTN1 | 2.63 | 2.25 | 1.46E-04 | 2.72E-02 |
| IGHGP | 2.64 | -0.07 | 4.04E-05 | 1.20E-02 |
| SCG3 | 2.66 | 0.52 | 1.31E-08 | 5.55E-05 |
| ALKAL1 | 2.70 | -0.81 | 1.72E-05 | 7.79E-03 |
| IGLV1-41 | 2.72 | -0.99 | 4.17E-06 | 2.79E-03 |
| APOBEC2 | 2.73 | 0.09 | 2.04E-07 | 3.15E-04 |
| S_HBV | 2.77 | 5.43 | 1.51E-04 | 2.75E-02 |
| AC113935.1 | 2.81 | -2.10 | 2.00E-07 | 3.15E-04 |
| ECEL1 | 2.82 | 0.61 | 1.14E-05 | 6.08E-03 |
| ABCC8 | 2.83 | 0.73 | 7.21E-06 | 4.10E-03 |
| IGLV3-9 | 2.90 | 1.76 | 4.23E-05 | 1.24E-02 |
| IL24 | 2.96 | -1.21 | 3.61E-07 | 4.29E-04 |
| INA | 3.00 | -0.97 | 7.83E-07 | 6.40E-04 |
| AC233755.2 | 3.07 | 1.61 | 5.21E-05 | 1.40E-02 |
| MTCO1P12 | 3.09 | 5.45 | 5.86E-06 | 3.56E-03 |
| CHGB | 3.18 | 1.03 | 2.87E-07 | 3.75E-04 |
| IGLV1-36 | 3.20 | 1.59 | 2.00E-05 | 8.43E-03 |
| REG4 | 3.22 | 1.59 | 5.71E-07 | 5.34E-04 |
| SLC8A2 | 3.27 | -0.15 | 3.43E-08 | 8.16E-05 |
| AMHR2 | 3.34 | -0.95 | 6.55E-08 | 1.22E-04 |
| CEL | 3.53 | 3.20 | 1.14E-08 | 5.55E-05 |
| MATN4 | 3.71 | -0.68 | 1.31E-09 | 1.15E-05 |
| PCSK1 | 3.74 | 1.48 | 2.11E-08 | 6.12E-05 |
| CALCA | 3.82 | 2.38 | 2.03E-08 | 6.12E-05 |
| LEFTY1 | 3.87 | 2.87 | 2.93E-09 | 1.91E-05 |
| GP2 | 4.05 | 5.12 | 9.45E-07 | 7.49E-04 |
| PAEP | 5.00 | 4.35 | 3.08E-08 | 8.05E-05 |
| AC107294.3 | 5.53 | 0.74 | 4.78E-12 | 1.25E-07 |

**Abbreviation:** CPM, counts per million; FC, fold change; FDR, false discovery rate.
