# Supplementary material for: Nucleotide variants in hepatitis B virus preS region predict the recurrence of hepatocellular carcinoma
Source: Aging (Albany NY). 2021 Sep 17;13(18):22256–75. doi: 10.18632/aging.203531 (PMC8507287; doi:10.18632/aging.203531)
Supplement: Supplementary Tables 7, 8 [file aging-13-203531-s004.pdf]

Supplementary Table 7. Transcription factor binding sites predicted in the region of preS2.

Motif ID	Transcription factor	Start	Stop	Strand	Score	*P* value	FDR
MA0484.1	HNF4G	138	152	-	8.38	2.25e-4	0.06
MA0505.1	Nr5a2	29	43	+	4.90	4.93e-4	0.07
MA0505.1	Nr5a2	40	54	-	3.34	7.89e-4	0.07
MA0505.1	Nr5a2	35	49	+	3.21	8.21e-4	0.07
MA1101.1	BACH2	30	43	-	6.69	3.69e-4	0.10
MA0114.3	Hnf4a	136	151	-	-4.58	4.23e-4	0.11
MA0501.1	MAF::NFE2	28	42	-	3.16	4.18e-4	0.12
MA1147.1	NR4A2::RXRA	35	49	-	5.77	5.69e-4	0.15
MA0150.2	Nfe2l2	32	46	-	3.61	7.08e-4	0.20
MA0728.1	Nr2f6(var.2)	27	41	+	-18.34	8.13e-4	0.23
MA0138.2	REST	135	155	+	-3.04	9.58e-4	0.23

Abbreviation: FDR, false discovery rate.

Supplementary Table 8. Gene sets enriched in the tumors with high/low frequencies of two HBV variants.

Gene set	Size	ES	NES	P value	FWER
**Gene sets enriched in the tumors with low frequencies of two HBV variants**					
CHIANG_LIVER_CANCER_SUBCLASS_PROLIFERATION_DN	178	-0.52	-2.73	<1E-3	<1E-3
CHIANG_LIVER_CANCER_SUBCLASS_CTNNB1_UP	170	-0.51	-2.66	<1E-3	<1E-3
CAIRO_HEPATOBLASTOMA_CLASSES_DN	213	-0.44	-2.35	<1E-3	0.003
LEE_LIVER_CANCER_SURVIVAL_UP	177	-0.44	-2.32	<1E-3	0.005
FARMER_BREAST_CANCER_CLUSTER_1	47	-0.52	-2.15	<1E-3	0.051
**Gene sets enriched in the tumors with high frequencies of two HBV variants**					
NIKOLSKY_BREAST_CANCER_8Q23_Q24_AMPLICON	145	0.62	2.86	<1E-3	<1E-3
CHIANG_LIVER_CANCER_SUBCLASS_PROLIFERATION_UP	174	0.56	2.62	<1E-3	<1E-3
SOTIRIOU_BREAST_CANCER_GRADE_1_VS_3_UP	152	0.52	2.41	<1E-3	<1E-3
BOYAULT_LIVER_CANCER_SUBCLASS_G3_UP	190	0.51	2.41	<1E-3	0.001
LEE_LIVER_CANCER_SURVIVAL_DN	174	0.51	2.39	<1E-3	0.001
KAMMINGA_EZH2_TARGETS	40	0.65	2.39	<1E-3	0.001
NIKOLSKY_BREAST_CANCER_8Q12_Q22_AMPLICON	122	0.54	2.38	<1E-3	0.001
NIKOLSKY_BREAST_CANCER_17Q11_Q21_AMPLICON	105	0.54	2.32	<1E-3	0.001
KOBAYASHI_EGFR_SIGNALING_24HR_DN	251	0.47	2.32	<1E-3	0.001
BURTON_ADIPOGENESIS_3	93	0.54	2.32	<1E-3	0.001
MITSIADES_RESPONSE_TO_APLIDIN_DN	247	0.47	2.28	<1E-3	0.001
ROSTY_CERVICAL_CANCER_PROLIFERATION_CLUSTER	140	0.50	2.27	<1E-3	0.001
SHEDDEN_LUNG_CANCER_POOR_SURVIVAL_A6	445	0.44	2.25	<1E-3	0.002
WONG_EMBRYONIC_STEM_CELL_CORE	332	0.45	2.24	<1E-3	0.002
BORCZUK_MALIGNANT_MESOTHELIOMA_UP	308	0.44	2.20	<1E-3	0.006
AGUIRRE_PANCREATIC_CANCER_COPY_NUMBER_UP	294	0.45	2.19	<1E-3	0.007
VILLANUEVA_LIVER_CANCER_KRT19_UP	167	0.46	2.16	<1E-3	0.01
CHIN_BREAST_CANCER_COPY_NUMBER_UP	23	0.68	2.16	<1E-3	0.01
CHIANG_LIVER_CANCER_SUBCLASS_UNANNOTATED_DN	192	0.46	2.15	<1E-3	0.011
RHODES_CANCER_META_SIGNATURE	65	0.54	2.15	<1E-3	0.011
ABRAMSON_INTERACT_WITH_AIRE	43	0.57	2.14	<1E-3	0.014
DAVICIONI_MOLECULAR_ARMS_VS_ERMS_DN	174	0.45	2.11	<1E-3	0.022
LI_WILMS_TUMOR_VS_FETAL_KIDNEY_1_DN	164	0.46	2.10	<1E-3	0.023
LE_EGR2_TARGETS_UP	107	0.48	2.09	<1E-3	0.029
REN_BOUND_BY_E2F	61	0.52	2.07	<1E-3	0.042
WOO_LIVER_CANCER_RECURRENCE_UP	103	0.48	2.07	<1E-3	0.043
PAL_PRMT5_TARGETS_UP	200	0.43	2.07	<1E-3	0.043
WAMUNYOKOLI_OVARIAN_CANCER_GRADES_1_2_UP	139	0.46	2.07	<1E-3	0.044
HEIDENBLAD_AMPLIFIED_IN_PANCREATIC_CANCER	55	0.53	2.07	<1E-3	0.047
BIDUS_METASTASIS_UP	210	0.43	2.06	<1E-3	0.051
JECHLINGER_EPITHELIAL_TO_MESENCHYMAL_TRANSITION_DN	56	0.53	2.06	<1E-3	0.052
WHITEFORD_PEDIATRIC_CANCER_MARKERS	113	0.47	2.06	<1E-3	0.052
JIANG_AGING_CEREBRAL_CORTEX_DN	45	0.55	2.06	1.55E-03	0.054
MEINHOLD_OVARIAN_CANCER_LOW_GRADE_DN	19	0.67	2.05	<1E-3	0.058
TURASHVILI_BREAST_LOBULAR_CARCINOMA_VS_DUCTAL_NORMAL_UP	66	0.51	2.05	<1E-3	0.059
GRAHAM_NORMAL_QUIESCENT_VS_NORMAL_DIVIDING_DN	87	0.49	2.05	<1E-3	0.061
NIKOLSKY_BREAST_CANCER_17Q21_Q25_AMPLICON	298	0.41	2.04	<1E-3	0.069
YAO_TEMPORAL_RESPONSE_TO_PROGESTERONE_CLUSTER_14	143	0.45	2.04	<1E-3	0.072
MARKEY_RB1_ACUTE_LOF_UP	229	0.42	2.04	<1E-3	0.074
DING_LUNG_CANCER_EXPRESSION_BY_COPY_NUMBER	100	0.47	2.03	<1E-3	0.076
CROONQUIST_IL6_DEPRIVATION_DN	95	0.47	2.03	<1E-3	0.076
HOSHIDA_LIVER_CANCER_SUBCLASS_S2	114	0.45	2.03	<1E-3	0.082
RHODES_UNDIFFERENTIATED_CANCER	69	0.50	2.03	<1E-3	0.085
VECCHI_GASTRIC_CANCER_ADVANCED_VS_EARLY_UP	169	0.43	2.02	<1E-3	0.088
BLUM_RESPONSE_TO_SALIRASIB_DN	336	0.40	2.02	<1E-3	0.091
FUJII_YBX1_TARGETS_DN	194	0.43	2.02	<1E-3	0.091
MARKEY_RB1_CHRONIC_LOF_UP	107	0.47	2.02	<1E-3	0.097

Abbreviation: ES, enrichment score; NES, normalized ES; FWER, familywise-error rate.
